# Desire for Birth Companionship Among Pregnant Women Attending Antenatal Care in Debremarkos City, Northwest Ethiopia: Magnitude and Associated Factors

**DOI:** 10.3389/fgwh.2022.823020

**Published:** 2022-04-07

**Authors:** Hussien Mohammed Assfaw, Mulunesh Abuhay, Melaku Hunie Asratie

**Affiliations:** ^1^Department of Clinical Midwifery, School of Midwifery, College of Medicine and Health Sciences, University of Gondar, Gondar, Ethiopia; ^2^Department of Women's and Family Health, School of Midwifery, College of Medicine and Health Sciences, University of Gondar, Gondar, Ethiopia

**Keywords:** antenatal care, companionship, Debremarkos, desire, Ethiopia

## Abstract

**Background:**

Birth companionship is one of the components of the respectful maternity continuum of care recommended by the World Health Organization (WHO). Women's desire for birth companionship needs to be given attention during the antenatal care period to make them ready during labor and delivery. There is a dearth of study about the status of women's desire for birth companionship and associated factors.

**Objective:**

This study aimed to assess the prevalence of desire for birth companionship and associated factors among pregnant women in Debremarkos city, northwest Ethiopia.

**Methods:**

Institution-based cross-sectional study was conducted from February 1, 2021 to March 30, 2021 in Debremarkos city, northwest Ethiopia. A total of 423 participants were accessed by systematic random sampling. A face-to-face interviewer-administered questionnaire was employed. The data were entered into Epi data version 4.6 and transformed to SPSS version 25. Binary logistic regression analysis was done, and variables with a *p*-value ≤ 0.2 on bivariable analysis were taken for multivariable analysis. Adjusted odds ratio with a 95% confidence interval was used to detect the association, and a *p*-value of <0.05 in the multivariable analysis was used to declare statistical significance.

**Results:**

The prevalence of desire for birth companionship was 57.45% (52.6–62.2%). Women who were the primary decision-maker for maternal health care services [adjusted odds ratio (AOR) =3.0; 95% CI 1.7–5.6], women with planned pregnancy (AOR = 2.0; 95% CI 1.0–3.9), women who have no bad obstetric history (AOR = 2.3; 95% CI 1.2–4.4), and women whose 1st antenatal care visit starts within the second trimester (AOR = 2.6; 1.6–4.4) were statistically significant with desire on birth companionship.

**Conclusions:**

Desire of pregnant women for birth companionship was high in this study. Improving women's decision-making power, emphasis on the type of pregnancy, obstetrical history, and early initiation of antenatal care visit were the suggested areas to increase the desire of women for birth companionship.

## Background

One of the efforts to reduce maternal, neonatal, and child mortality increases the coverage of maternal health care services (1). As the evidence shows over the past decade, the coverage of maternal health care services has improved. As antenatal care had reached 62%, health facility delivery had reached 26% and postnatal care had reached 17 (2). With this improvement in the magnitude of health care services across the continuum of care from time to time, emphasis is shifting toward the quality of health care services that are delivered to the mother and the neonate (3). Specifically, improvement in the magnitude of health facility delivery and delivery attended by skilled health care provider needs great attention on the effect of client-centered health care provision, retention, quality, equity, and dignity of the laboring mother (4). A laboring mother is considered as she is at a period of extreme stress, anxiety, and fear in her life (5). During this situation, she needs supportive care that is derived from her social support (6). Emotional and social support of women's choice is core to the experience of care and to achieve positive person-centered health outcomes (7). Thus, World Health Organization (WHO) recommends that every woman is offered the option to experience labor and childbirth with a companion of her choice (8).

Birth companionship is a health care support given for a laboring mother that is provided by a family member, a partner, friend, doula, or health care professional. A woman has continuous tangible, informational, and social support from their preferred social network (9). The term birth companionship is expressed in different ways, which includes companion of choice at birth, birth companion, labor companion, social support during labor and delivery, emotional support during birth, supportive companionship, and continuous support for women during childbirth (9–12). But continuous support during childbirth is the most recommended way of expression (13). Supporting the strategy for the presence of women's companion of choice during labor and delivery is a well-evaluated intervention with concerning respecting women's autonomy and interest and can be the agent for the improvement in the quality of care during labor and child birth (14).

Having a labor companion provides multiple benefits and is been recommended by WHO (15). There is evidence that shows the positive pregnancy outcome for mother and baby among those laboring mothers with labor companionship (10, 15). Birth companions also have great importance for health care providers when under staffs facilities by doing different activities such as going to purchase supplies and drugs, facilitation of delivery process by helping women to and from the delivery bed (16–18). The most common reasons women cited for wanting a companion were to have someone to attend to their needs and help them make decisions (19). Most of the women who had support from those companions were less likely to use obstetric analgesia to relive labor pain, less likely to have cesarean delivery, and less likely to deliver by instrumental (20). A study done in idea shows that 42, 21, 12, and 7% of laboring women relieve from psychological disturbance, from the intensity of labor pain, from apprehension, and have a feeling of strength and encouragement, respectively (21). There is a piece of supportive evidence that shows women with a birth companion have a smooth delivery process and are highly satisfied with their childbirth process (22).

A readily available supportive person needs to fulfill the needs of a laboring mother. The additional laboring mothers often felt helpless during labor and delivery and they were considered as providers who were not with them all the time (20, 23). In other cases, health care providers are usually too much busy with additional things and ensure that when a laboring woman need help, there is someone readily available to help them, like to call the provider if they developed a problem, need to go to the bathroom, and when she went into the second stage of labor (24, 25).

Even though all the above-listed engagements of birth companions during labor and delivery, most were not allowed in the ward to cascade such activities. Evidence done in different areas had shown the low prevalence of birth companionship, Arba Minch Ethiopia (13.8%) (12), Kenya (29%) (9), and Nepal (19%) (14). The most common reasons reported by the kinds of literature for this low prevalence of birth companionship are health care providers distrust of companions, the situation that has been there in the ward like ward setup and privacy, and a lack of confidence in them in the event of complication (26, 27). The evidence done in Ethiopia shows that provider denial, institutional not allowed, and a lack of support accounts for 47.9, 21.1, and 2.0%, respectively, are the reasons for not having a companion during delivery (12). But the first thing that we have to prioritize is that respecting the woman's desire during the process of labor and delivery for the best outcome for both the woman and the newborn as well.

Giving attention to women's desire during the provision of maternity continuum of care is the best approach in order to achieve the sustainable development goal (28). There is evidence that shows the success of labor and delivery was good by respecting women's desire for her position during labor and delivery (29). After all, keeping women's desire during labor and delivery at the optimal label is mandatory. One of the women's desires that needs attention as it is recommended by the WHO is the “desire for birth companionship”. Despite all the aforementioned clarities about birth companionship which can be achieved whenever their desire is respected, there is limited evidence that shows the status of women's desires about birth companionship. The different studies have been done about the status of birth companions among postpartum period (12, 30), rather the desire of pregnant women about birth companionship was not yet studied in Ethiopia.

There is a need to deal with their desire for birth companionship during their antenatal care period as it is important for giving recommendations to make conducive environment during labor and delivery. From unpublished document, the current labor ward policy in Debremarkos city health facilities allows companions to stay with the laboring women continuously until the end of labor and delivery process. But companions ordered to get out of the ward while health care providers examine the laboring women. This makes discomfort on both the laboring women and the companion. Based on the finding of this study, the rules and regulations in general labor room policies will be re-adjusted to accommodate or in favor of companions based on parturient desire (21). Therefore, this study was aimed to assess the desire of pregnant women for birth companionship and associated factors among women attending antenatal care in Debremarkos city, northwest Ethiopia.

## Methods

### Study Design, Area, and Period

An institution-based cross-sectional study was conducted from February 1, 2021 to March 30, 2021 in Debremarkos city, northwest Ethiopia.

Debremarkos city is located in the East Gojjam Zone of the Amhara Region. Based on the population projection from 2014 to 2017 at all woreda levels estimated the population to be 92,470. Among the total projected population, 46,738 were women (31). Debremarkos city has totally seven kebeles and 24,914 households. The total number of health facilities in Debremarkos city is 25, of those there is one referral hospital, seven private clinics, three public health facilities, and fourteen health posts. All public health facilities and three private clinics can give antenatal care services. From monthly reports of health facilities, there are 2,000 pregnant women who attend antenatal care.

### Source Population

All pregnant women attending antenatal care in Debremarkos city public health facilities.

### Study Population

All pregnant women attending antenatal care in Debremarkos city public health facilities during the data collection period.

### Inclusion Criteria

All pregnant women attending antenatal care in public health facilities of Debremarkos city during the data collection period.

### Sample Size Determination

The sample size was calculated using the single population proportion formula by considering that the prevalence of desire for labor companionship in Debremarkos city among pregnant women attending antenatal care was estimated to be 50% as there was no study yet. By taking a non-response rate of 10%, and a margin of error of 5% with a 95% confidence interval, the sample size has been calculated as follows.


$$\text{n}=\frac{{\left(\text{z }\alpha /2\right)}^{2}\text{ p }(1-\text{p})}{{\text{d}}^{2}}=\frac{{\left(1.96\right)}^{2}0.5(0.5)}{0.05^{2}}\;=\;384$$

where *n*= estimated sample size, zα/2 = 1.96 the value given from the table 95% confidence interval, *p* = estimated proportion of desire for labor companionship.

After adding a non-response rate of 10%, the final estimated sample size for this study was 423.

### Sampling Technique and Procedure

Systematic random sampling was employed to take the desired sample size of the study participants. First, there is one referral hospital in Debremarkos city and taken as our study without inconsideration to sampling technique, and then, there is a shortlist of three public health facilities, of those two public health facilities were taken by simple random sampling technique. After selecting public health facilities, we had used a systematic random sampling technique by calculating “K” from the monthly reported number of antenatal care attendees and the calculated sample size. *K* = *N*/*n* where *N* = 2,000, *n* = 423 and *K* = 5.

The first participant was selected by simple random sampling technique from the value of k which is from 1 up to 5, and the second antenatal care attendee was our first participant. Every 5 antenatal care attendees were selected as study participants, and finally, we had taken a total of 423 antenatal care attendees asked about their desire for labor companionship and associated -factors.

### Study Variable

Desire for birth companionship, sociodemographic, obstetrical, maternal health care services, and health facility were the variables used in our study.

#### Desire for Birth Companionship

The dependent variable was measured by Yes/No. Antenatal care attendee pregnant women were asked about their desire of labor companionship “Have you a desire for labor companionship while you are in labor?”

#### Birth Companionship or Utilization of Labor Companion

Women have a continuous emotional, tangible, informational, and social support with a preferred companion from their social networks during labor in the labor ward (7).

### Data Collection Instrument and Procedure

A structured interviewer-administered questionnaire was used. The tool was adapted from different kinds of literature. The questionnaire was first prepared in Amharic and translated to English by experts in both English and the subject matter in obstetrics and gynecology then back to the Amharic language which is preferable to the local language of the study participants.

The questionnaire was pretested at Bahir Dar public health facilities with a sample size of 5% (22). During the process of the pretest appropriateness of the questionnaire and clarity in language for the study, participants were checked.

A face-to-face interview was employed to collect the necessary data. The data were collected by 5 BSc midwifery health care practitioners and 2 BSc midwifery health care providers who were involved in the supervision of the data collection.

### Data Processing and Analysis

Data were entered using Epi data version 4.6 and exported into SPSS version 25 for the analysis. Data were checked for completeness, and coded and descriptive statistics were done like frequency, percent, mean, and standard deviation. Finally, the data were presented with text, table, and graph.

A binary logistic regression model was fitted to determine the association between the independent variables and the outcome variables. Bivariable and multivariable analyses were done. All variables with a *p*-value of ≤0.2 from the bivariable analysis were used for multivariable analysis. An adjusted odds ratio with a 95% confidence interval was used to declare the statistical significance of the variable. A variable with a *p*-value of <0.05 in the multivariable analysis was considered statistically significant with the outcome variable desire for labor companionship.

## Results

### Sociodemographic Characteristics of Participants and Their Husband in Debremarkos City, Northwest Ethiopia

Among all study participants, 179 (42.3%) of them were within the age limit of 20–34 years. The mean age of the study participants was 30.52 years with a standard deviation of 7.26 years. A total of 337 (79.7%) of the participants were orthodox, 320 (75.7%) of them were married, and 238 (56.3%) of the participants have had no formal education. Of all study participants, 273 (64.5%) resided in the rural part, 180 (42.6%) of the women were farmers by occupation, and 196 (46.3%) of women's husbands were had no formal education. Their median monthly income was 2,400 EB with (IQ R of 1900-3286EB) (Table 1).

**Table 1:** Sociodemographic characteristics of women in Debremarkos city, northwest Ethiopia.

**Characteristics**	**Frequency**	**Percent**
**Age of women**		
15–19	104	24.6
20–34	179	42.3
35–49	140	33.1
**Religion**		
Orthodox	337	79.7
Muslim	63	14.9
^Other*^	23	5.4
**Marital status**		
Married	320	75.7
Cohabiting	41	9.7
^Other**^	62	14.6
**Educational status of women**		
Have no formal education	238	56.3
Primary (grade 1–8)	49	11.6
Secondary (grade 9–12)	63	14.9
College and above	73	17.2
**Residency**		
Rural	273	64.5
Urban	150	35.5
**Exposure to any kind of media**		
Yes	228	53.9
No	195	46.1
**Occupational status of the women**		
Farmer	180	42.6
House wife	80	18.9
Government employee	63	14.9
^Other***^	100	23.6
**Educational status of the husband**		
Have no formal education	196	46.3
Primary (grade 1–8)	21	5
Secondary (grade 9–12)	60	14.2
College and above	146	34.5
**Average family monthly income**		
≤1,199	3	0.7
12,000–2,499	187	44.2
≥2,500	233	55.1

Other*: *Catholic, protestant;*

Other**: *Single, widowed;*

Other***: *Daily laborer, student*.

### Health Care Service-Related Characteristics of Women

Among all respondents, 202 (47.8%) of pregnant women can decide about maternal health care services independently, 208 (49.2%) of them can decide independently to have access to family planning services, and 292 (60%) of the participants were reported as they cannot easily access ambulance services (Table 2).

**Table 2:** Health care service-related factors of pregnant women in Debremarkos city, northwest Ethiopia.

**Characteristics**	**Frequency**	**Percent**
**Decision-maker for attending maternal health care services**		
Independently by women	202	47.8
Husband/partner	30	7
Joint with other	191	45.2
**Decision-maker for family planning**		
Independently by women	208	49.2
Husband/partner	178	42.1
Jointly with others	37	8.7
**Availability of ambulance services**		
Yes	131	40
No	292	60

### Obstetrical Related Characteristics of Pregnant Women

Of all respondents, 243 (57.5%) of the women were gravida five and above, and 235 (55.4%) of the participants their current pregnancy was planned. A total of 383 of the participants were not developed any kind of obstetrical complication during the current pregnancy and 301 (71.2%) of the respondents were have no bad obstetric history (Table 3).

**Table 3:** Obstetrical related factors of pregnant women in Debremarkos city, northwest Ethiopia.

**Characteristics**	**Frequency**	**Percent**
**Gravidity**		
1–2	67	15.8
3–4	113	26.7
≥5	243	57.5
**Current pregnancy planned**		
Yes	235	55.4
No	188	44.4
**Complication during the current pregnancy**		
No	383	90.5
Yes	40	9.5
**Bad obstetric history**		
No	301	71.2
Yes	122	28.8

### Maternal Health Care Service-Related Factors of Pregnant Women

From 423 respondents, 257 (60.8%) of them their 1st antenatal care was started within the second trimester, 368 (87%) of them have said that there was health care provider support during the provision of antenatal care, 326 (77.1%) of them were with antenatal care visit of 1st to 3rd, and 233 (55.1%) of them were attended their antenatal care from the hospital. Among all participants, 216 (51.1%) had no privacy during antenatal care services, and 417 (98.6%) of were had no exposure to a traditional birth attendant (Table 4).

**Table 4:** Maternal health care service-related factors of pregnant women in Debremarkos city, northwest Ethiopia.

**Characteristics**	**Frequency**	**Percent**
**Gestational age for 1st antenatal care visit**		
Within second trimester	257	60.8
Above second trimester	166	39.2
**Health care provider support during antenatal care**		
Yes	368	87
No	55	13
**Number of antenatal care**		
1st to 3	326	77.1
4th and above	97	22.9
**Site of antenatal health care services**		
Hospital	233	55.1
Health center	190	44.9
**Privacy during antenatal care services**		
Yes	207	48.9
No	216	51.1
**Experience for traditional birth attendant**		
Yes	6	1.4
No	417	98.6

### The Prevalence of Desire for Labor Companionship Among Pregnant Women Attending Antenatal Care in Debremarkos City, Northwest Ethiopia

**Figure 1:**
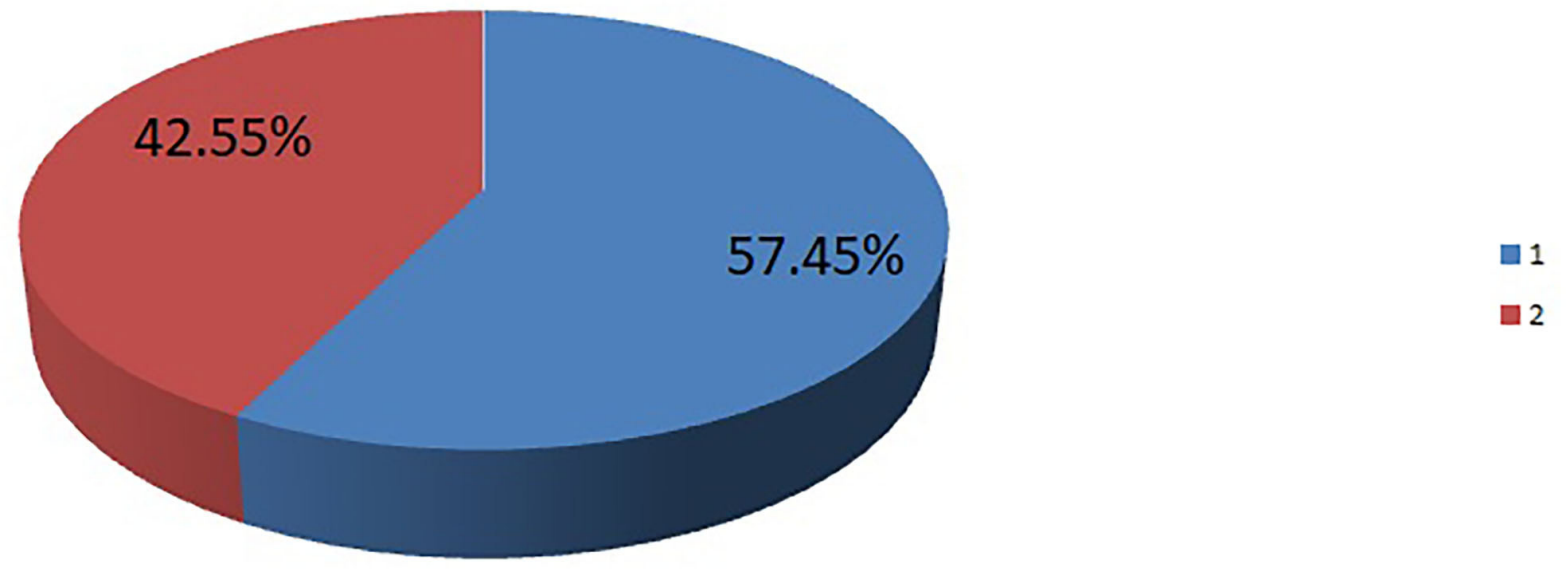
The prevalence of pregnant women desired for birth companionship in Debremarkos city, northwest Ethiopia.

The magnitude of pregnant women desired for labor companionship in this study was found to be 57.45% (52.6–62.2%) (Figure 1).

### Factors Associated With Pregnant Women's Desire for Birth Companionship in Debremarkos City, Northwest Ethiopia

From bivariable analysis, six sociodemographic variables (age of the pregnant women, religion, marital status, educational status of the women, residency, and exposure to any type of media) and other eight variables were significantly associated with the outcome variable desire for birth companionship.

From multivariable analysis, four variables, decision-maker for maternal health care services, current pregnancy type whether it was planned or not, bad obstetric history of the women, and time of 1st antenatal care visit start, were continued significantly with the outcome variable desire for birth companionship.

Women who were the decision-makers independently to attend maternity continuum of care 3.0 (1.7–5.6) times more likely desired for birth companionship as compared to those women the decision was made jointly with others. Respondent whose current pregnancy was planned 2.0 (1.0–3.9) times more likely desired for birth companionship as compared to women with current pregnancy was not planned. Women without bad obstetric history 2.3 (1.2–4.4) times more likely desired birth companionship as compared to those with bad obstetric history. The participant who has started their 1st antenatal care visit within the second trimester was 2.6 (1.6–4.4) times more likely desired to birth companionship as compared to women who have started their antenatal care above the second trimester (Table 5).

**Table 5:** Factors associated with women's desire for birth companionship in Debremarkos city, northwest Ethiopia.

	**Desire for birth companion**		**COR(95% CI)**	**AOR(95% CI)**
**Characteristics**	**Yes**	**No**		
**Age of the women**				
15–19	34	70	0.5 (0.3–0.87)**	N/A
20–34	78	101	0.8 (0.5–1.3)	N/A
35–49	68	72	1	1
**Religion**				
^Other*^	6	17	0.4 (0.16–0.81)	N/A
Muslim	24	39	0.76 (0.4–1.3)	N/A
Orthodox	150	187	1	1
**Marital status**				
Marred	164	156	0.3 (0.19–0.6)	N/A
Cohabiting	33	8	1.4 (0.16–2.14)	N/A
^Other**^	46	16	1	1
**Educational status of the women**				
Have no formal education	130	108	3.9 (2.1–7.2)	N/A
Primary (grade 1–80	19	30	2.1 (0.9–4.6)	N/A
Secondary (grade 9–12)	14	49	0.9 (0.4–2.1)	N/A
College and above	17	56	1	1
**Residency**				
Rural	145	128	3.7 (2.3–5.8)	N/A
Urban	35	115	1	1
**Exposure to any type of media**				
Yes	121	107	2.6 (1.7–3.8)	N/A
No	59	136	1	1
**Decision-maker for maternal health care services**				
Independently by women	153	49	5.5 (3.6–8.5)^***^	3.1 (1.7–5.6)^***^
Husband/partner	21	9	4.1 (0.8–5)	0.8 (0.3–2.5)^***^
Jointly with other	69	122	1	1
**Time to reach health facility**				
≤30 min	65	159	0.22 (0.2–0.4)**	N/A
>30 min	115	84	1	1
**Availability of ambulance services**				
Yes	81	50	3.1 (2.1–4.8)	N/A
No	99	193	1	1
**Current pregnancy planned**				
Yes	168	67	3.8 (2.5–5.7)^***^	2.0 (1.0–3.9)^***^
No	75	113	1	1
**Complication during current pregnancy**				
No	212	171	0.36 (0.1–0.78)	N/A
Yes	31	9	1	1
**Bad obstetric history**				
No	182	119	1.6 (1.0–2.4)**	2.3 (1.2–4.4)^***^
Yes	60	61	1	1
**Decision-maker for family planning**				
Independently by women	151	57	2.0 (0.9–4.1)	N/A
Husband/partner	107	71	2 (1–3.1)	N/A
Jointly with other	16	21	1	1
**1st ANC start gestational age**				
Within second trimester	181	76	3.9 (2.6–6.0)^***^	2.6 (1.6–4.4)^***^
Greater than second trimester	62	104	1	1

*AOR, adjusted odds ratio; CI, confidence interval; COR, crude odds ratio; N/A, not associated;*

other*: *Catholic, protestant;*

other**: *single, separated;*

*** *p-value < 0.05*.

## Discussion

The coverage of maternal health care services has improved from time to time. Currently, it is time to give emphasis on the quality of the services across the continuum of care. Health facility delivery is one component of the maternity continuum of care, and its quality can be achieved by a multiphasic approach either from the laboring mother or from the health care provider and health facility perspective.

Currently, WHO recommends laboring women can have a birth companion while she comes to the health facility for giving birth. It also clearly states the advantage of birth companions for the improvement in the quality of the service provided at the health facility.

Exploring the desire of pregnant women for birth companionship for their future labor and delivery is very important in order to make the health facility ready to accommodate those companions, and even it is important to create a positive impression on health care providers about those companions.

Despite those advantages, there is no study done yet about the desire of pregnant women on their choosing birth companions. Therefore, this study finds out the prevalence of desired pregnant women about birth companionship during labor and delivery in Debremarkos city, northwest Ethiopia.

The prevalence of desired women for labor companionship during labor and delivery in this study is 57.45%. This finding is slightly higher than the estimated prevalence for the sample size calculation which was 50%. The possible explanation for this higher desire for birth companionship during labor and delivery in this study might be that the majority of women in our study had no formal education and those participants need the nearest person for communication-related health facility services. There is evidence that shows women who live in the marginalized area of Ethiopia with limited educational level are highly experiencing traditional birth attendants, and even they need to make them as companion at the health facility (32, 33). The finding of this study also higher than study conducted in Arba Minch Ethiopia (43.7%) (12), Kenya (37%) (9), Nigeria (22.1%) (34), and Tanzania (44.7%) (11). The possible explanation for higher magnitude in this study compared to the study conducted at Arba Minch Ethiopia might be due to the variation in the study population. Collecting the data among pregnant women for their future desire of labor companionship in our study might make inflated magnitude compared to study done among postpartum period women. The difference with the study done in Nigeria might be due to the study setting in this study that includes both health centers and hospitals whereas the former study only includes participants from hospitals. Most of the time health care provider at the hospital level does not allow visitors, due to over crowdedness (35). Therefore, women might adapt alone to accessing health care services during antenatal care and they might not be desired to have a companion during labor and delivery. The study also conducted in Kenya was a retrospective which depended on postpartum period women 9 weeks passed before the survey, and this might make lower the magnitude compared to this study conducted among pregnant women attending antenatal care.

Regarding the factors associated with the desire for birth companionship among pregnant women attending antenatal care decision-maker to attend maternal health care services, current pregnancy was planned, bad obstetric history, and gestational age at 1st antenatal care visit started were the variables significantly associated.

Women who were the independent decision-maker to attend maternal health care services were 3.1 times more desired for birth companionship compared to deciding jointly with others. This finding is supported by a study done in Kenya (36). The possible explanation could be that women who have decision-making power on the maternity continuum of care also might have the possibility to decide about the supporter person at the time of delivery. On the other hand, independent decision-makers about accessing maternal health care services are mostly familiar with the condition of the health facility and they are well-informed about the advantage of attendants during labor and delivery in addition to health care provider's efforts. This idea is supported by the evidence done at different areas, Pakistan (37), Bangladesh (38), Ghana (39), and Indonesia (40).

Women with current pregnancy planned 2.0 times more desired for birth companionship as compared to current pregnancy was not planned. The possible explanation could be that women with planned pregnancy are mostly preoccupied with over care of the fetus starting from conception to the expected date of delivery (41). One of the characteristics displayed by the majority of women with a planned pregnancy is that they need to have a supportive person throughout their pregnancy and even at the time of labor and delivery (42, 43). On the other hand, those women with an unplanned pregnancy are less likely to give care about their pregnancy (44, 45), even they are not happy to disclose their pregnancy status for the others, and they need to bear alone (46, 47).

Women had no bad obstetric history 2.3 times more desired for birth companionship compared to women with bad obstetric history. The possible explanation might be due to women with no bad obstetric history mostly cautious about their pregnancy and highly adhered to the maternity continuum of care (48). Birth companionship is one of the recommended interventions by the WHO and sermonized by health care providers during the antenatal care period (8). Therefore, the more adhered to antenatal care visits the more positive impression about birth companionship and become the more desired to have during labor and delivery.

Participants' antenatal care starts within the second trimester 2.6 times more desired for birth companionship compared to women who start antenatal care above the second trimester. Antenatal care is the noteworthy predictor of pregnancy, and it has a multidimensional impact on the positive outcome of the women and the delivered baby (49). Most of the women who had initiated antenatal care lately in Ethiopia were preoccupied with traditional beliefs of disclosing pregnancy might expose them for harms, and this attitude also has been there during labor and delivery (50, 51). Due to this reason, women do not need to have a birth companion during labor and delivery. On the other hand, a lack of attending full components of antenatal care might be the factor for those low desired for birth companionship, as antenatal care is very important to have plenty of information about birth companionship (52, 53).

## Conclusions

The magnitude of desire for birth companionship in the study area was found to be high. Independent decision-making power to attend maternal health care services, planned pregnancy, have no bad obstetric history, and antenatal care starts within second trimester were the variables significantly associated with desire for birth companionship.

## Data Availability Statement

The original contributions presented in the study are included in the article/supplementary material, further inquiries can be directed to the corresponding author/s.

## Ethics Statement

Ethical review and approval was not required for the study on human participants in accordance with the local legislation and institutional requirements. Written informed consent for participation was not required for this study in accordance with the national legislation and the institutional requirements.

## Author Contributions

All authors made a significant contribution to the work reported, whether that is in the conception, study design, execution, acquisition of data, analysis and interpretation, or in all these areas, took part in drafting, revising, or critically reviewing the article, gave final approval of the version to be published, have agreed on the journal to which the article has been submitted, and agreed to be accountable for all aspects of the work.

## Conflict of Interest

The authors declare that the research was conducted in the absence of any commercial or financial relationships that could be construed as a potential conflict of interest.

## Publisher's Note

All claims expressed in this article are solely those of the authors and do not necessarily represent those of their affiliated organizations, or those of the publisher, the editors and the reviewers. Any product that may be evaluated in this article, or claim that may be made by its manufacturer, is not guaranteed or endorsed by the publisher.

## Abbreviations

AOR: adjusted odds ratio
CI: confidence interval
COR: crude odds ratio
IRB: institutional review board
N/A: not associated
WHO: World Health Organization.
